# Prediction of Nursing Home Admission Using the FRAIL-NH Scale Among Older Adults in Post-Acute Care Settings

**DOI:** 10.1007/s12603-023-1893-1

**Published:** 2023-03-10

**Authors:** M. Yasuoka, M. Shinozaki, K. Kinoshita, J. Li, M. Takemura, A. Yamaoka, Y. Arahata, I. Kondo, H. Arai, Shosuke Satake

**Affiliations:** 1 Department of Frailty Research, Research Institute, National Center for Geriatrics and Gerontology, 7-430 Morioka, Obu, Aichi, 474-8511 Japan; 2 Department of Sleep-Wake Disorders, National Center of Neurology and Psychiatry, National Institute of Mental Health, Kodaira, Japan; 3 Department of Neurology, Hospital, National Center for Geriatrics and Gerontology, Obu, Aichi, Japan; 4 Center for Frailty and Locomotive Syndrome, National Center for Geriatrics and Gerontology, Obu, Aichi, Japan; 5 Hospital, National Center for Geriatrics and Gerontology, Obu, Aichi, Japan; 6 National Center for Geriatrics and Gerontology, Obu, Aichi, Japan; 7 Department of Geriatric Medicine, Hospital, National Center for Geriatrics and Gerontology, Obu, Aichi, Japan

**Keywords:** Frail, post-acute care setting, older adults, institutionalization

## Abstract

**Objectives:**

The FRAIL-NH scale was developed to identify frailty status in nursing home residents. The purpose of this study was to examine the utility of the FRAIL-NH scale for predicting nursing home admission among patients in post-acute care settings.

**Design/Setting/Participants:**

This single-center, prospective, observational cohort study included participants aged 65 years or older who were admitted to a community-based integrated care ward (CICW) between July 2015 and November 2020.

**Measurements:**

Using the CICW database, we retrospectively classified participants as robust, prefrail, or frail based on the FRAIL-NH scale the score by identifying variables from our database that were most representative of each component. The following data were collected: examination findings, CICW admission and discharge information, length of CICW stay, and nursing home admission. The participants were divided into two groups based on whether or not they were admitted to a nursing home after CICW discharge. The hazard ratios (HRs) and 95% confidence intervals (CIs) for nursing home admission were calculated according to the FRAIL-NH categories using the Cox proportional hazards models with reference to the robust group. In the multivariate adjusted model, we adjusted for age, sex, nutritional status, cognitive function, living status, and economic status.

**Results:**

Data of 550 older adults were analyzed, of which 118 were admitted and 432 were not admitted to a nursing home. The frail group had a higher risk of nursing home admission (HR, 2.22; 95% CI 1.32–3.76) than the robust group.

**Conclusions:**

This study showed that the FRAIL-NH scale was beneficial for predicting nursing home admission among older adults in the post-acute care setting. Thus, assessment using the FRAIL-NH scale may help to consider preparation and support for life after discharge.

**Electronic Supplementary Material:**

Supplementary material is available for this article at 10.1007/s12603-023-1893-1 and is accessible for authorized users.

## Introduction

**O**lder adults, particularly frail older people, may have difficulty returning to their previous living place following hospitalization due to acute illness because of decline in activities of daily living (ADLs) (1). Therefore, obtaining more detailed information about the possibility of returning home during hospitalization may help medical staff and care professionals set more realistic goals of rehabilitation and prepare appropriate services for older patients after discharge (2).

The frailty phenotype and frailty index represent definitions of frailty in older adults (3, 4). Morely et al. developed a simplified and validated frailty screening tool, FRAIL scale, which measured fatigue, resistance, ambulation, illnesses, and loss of weight (5). Although the frailty phenotype model is useful for assessing physical frailty in independent older adults, it may be difficult to accurately assess the frailty status in older adults with mild to moderate decline in ADLs after acute illness (6). Using the phenotype frailty model, the prevalence of frailty in nursing homes was much higher than those reported in community studies (7). The available frailty phenotypes were difficult to identify for nursing home residents. Therefore, Morley et al. developed the FRAIL-NH scale to assess the frailty status in nursing home residents who have some difficulties with ADLs but who, with appropriate treatment, have the potential to recover and improve their outcomes (8). The FRAIL-NH scale is a 7-item screening tool. It includes domains related to potentially reversible variables including: fatigue, resistance, ambulation, incontinence or illness, loss of weight, nutritional approach, and help with dressing. The FRAIL-NH scale includes characteristics of the frailty phenotype model and classification systems based on the frailty index (3, 9). It was demonstrated to be useful for the prediction of mortality (10), hospitalization (11), and falls (11) among nursing home residents (12).

Community-based integrated care wards (CICWs), established in Japan since 2014, are intended to provide post-acute care with a focus on treatment at home and to coordinate services and rehabilitation opportunities for daily living after discharge. This is an intermediate stage between the hospital and patients’ house. In the US, this would represent a “skilled rehabilitation facilities”. In general, patients in CICWs are of older age and have reduced ADLs compared with those in general wards (13). To date, there is little evidence on whether the FRAIL-NH score could predict the likelihood of CICW patients returning home or moving to a nursing home.

Therefore, the purpose of this study was to examine the utility of the FRAIL-NH scale for predicting nursing home admission among older adults in post-acute care settings. Our hypothesis is that the high FRAIL-NH score, which was developed for nursing home residents, predicts nursing home admission among patients in post-acute care settings.

## Methods

### Study Design and Participants

This single-center, prospective, observational cohort study included patients treated at the CICW of National Center for Geriatrics and Gerontology in Japan between July 2015 and November 2020. This registry was completed in November 2020 because CICW was converted to a care ward for patients with COVID-19. Written informed consent was obtained from all patients or their family members, as appropriate. Ethical approval was obtained from the relevant Ethics Committee of Human Research of the National Center for Geriatrics and Gerontology, Japan (No. 830).

Participants registered in the CICW database sequentially during the study period were retrospectively screened. The database was developed for a registry study that focused to clarify the association between frailty and home admission. The database contained information of participants with informed consent and those who were not planned to be discharged from the CICW within 2 weeks, were not in the terminal stage of life, or did not have a pacemaker. The CICW database included the information regarding skeletal muscle mass by using bioelectrical impedance analysis (BIA). We excluded patients having a pacemaker because BIA can cause interference with the pacemakers.

**Figure 1:**
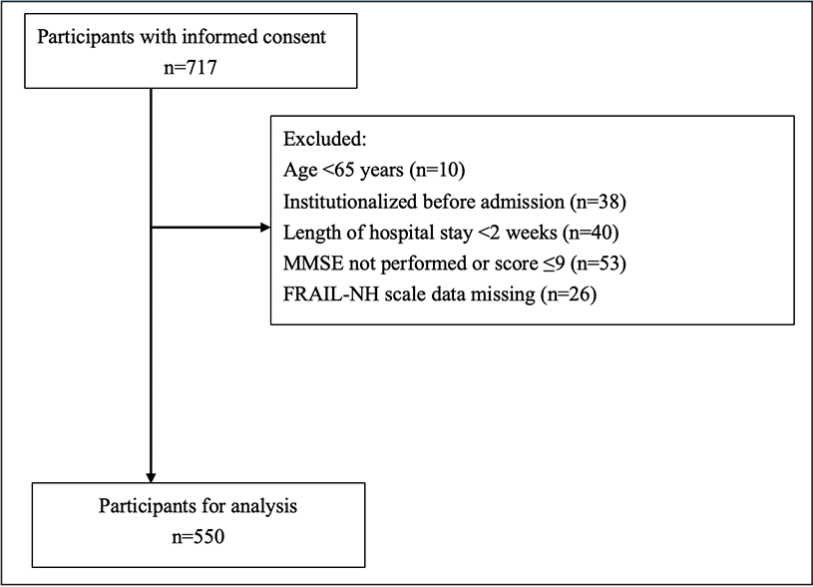
Flowchart of inclusion and exclusion for this study

The exclusion criteria of this research were visualized in Figure 1 and were as follows: (1) age under 65 years, (2) living in nursing homes before CICW admission, (3) length of hospitalization of less than 2 weeks, (4) Mini-Mental State Examination (MMSE) score not performed or of 9 or less, (14) and (5) missing measurements. Missing items of MMSE were replaced to 0, because these missing data represented the lacked ability to finish the item (e.g., fracture of the dominant hand, visual impairment or disturbance of consciousness). Of the screened 717 participants, 167 were excluded due to age under 65 years (n=10), living in a nursing home before CICW admission (n=38), CICW stay of less than 2 weeks (n=40), MMSE not performed or MMSE scores ≤9 (n=53), and missing data for Geriatric Depression Sacle 15 (GDS15) or the Mini Nutritional Assessment-Short Form (MNA-SF) or the Functional Independence Measure (FIM) completing all FRAIL-NH components (n=26). Finally, 550 older adults (258 with robust, 97 with prefrail, and 195 with frail status) were included in the analysis.

### FRAIL-NH Scoring

Using a standardized approach, we retrospectively calculated the FRAIL-NH scores by identifying variables from our database that were most representative of each component of the FRAIL-NH scale.

We used GDS15 (15), a part of FIM (16), a part of MNA-SF (17), and Functional Oral Intake Scale (FOIS) (18). GDS15 is a reliable and valid screening tool for depression among older adults (19). Answers are in the form of “yes” or “no” for 15 questions. The FIM consists of 13 motor and 5 social-cognitive items, assessing self-care, sphincter, management, transfer, locomotion, communication, social interaction, and cognition. All items are scored on 7-point ordinal scale with 7 reflecting complete independence, 6 modified independence, 5 supervision or set up, 4 minimal contact assistance or the subject expends >75% of the effort, 3 moderate assistance or the subjects expends 50 to 74% of the effort, 2 maximal assistance or the subject expends 25 to 49% of the effort, and 1 total dependence. MNA-SF contains 6 questions regarding food intake, involuntary weight loss, mobility, recent psychological stress or acute disease, neuropsychological problem, and calf circumference. The range of score was between 0 and 14 points. Weight loss during the last 3 months was asked (0: weight loss greater than 3 kg, 1: does not know, 2: weight loss between 1 and 3 kg, and 3: no weight loss) (17). FOIS is a 7-point ordinal scale most frequently used for the evaluation of functional oral intake by dysphagia patients. It assesses the oral intake of food and liquids (18). Level 1 represents nothing by mouth. Level 2 represents tube dependent with minimal attempts of food or liquid. Level 3 represents tube dependent with consistent oral intake of food or liquid. Level 4 represents total oral diet of a single consistency. Level 5 represents total oral diet with multiple consistencies, but requiring special preparation or compensations. Level 6 represents total oral diet with multiple consistencies without special preparation but with specific food limitations. Level 7 represents total oral diet with no restrictions.

Scoring of FRAIL-NH was completed by referring strictly to the following variables: GDS15 for Fatigue; Bed, Chair, and Wheelchair Transfer in FIM for Resistance; Walking and Wheelchair in the FIM for Ambulation; Bladder or Bowel in the FIM for Inconsistence; Weight loss over 1 kg during the last 3 months in MNA-SF for Weight Loss; FOIS for Nutritional Approach; and Dressing Upper or Lower Body in the FIM for Help with Dressing (Table 1). GDS15 scores: ≤5 as normal for “No,” 6–9 as minor depressive disorder for “Yes,” and ≥10 as major depressive disorders for “Patient health Questionnaire (PHQ-9) ≥10” (20). Regarding the MNA-SF, this tool comprises four categories of weight loss: no weight loss, weight loss between 1 and 3 kg, weight loss greater than 3 kg, and does not know. As previous studies have shown that the mortality was high among the last three categories with reference to the “no weight loss” category (21, 22), we assigned a score of 1 to these three categories and a score of 0 to the “no weight loss” category. FOIS scores Level 7 for “0”, Levels 4–6 for “1” and Levels 1–3 for “2”. The FRAIL-NH score was calculated as the sum of the scores in the seven components. Based on this score, the frailty status was categorized as follows: robust (0–5), prefrail (6–7), or frail (8–13) (23).

**Table 1:** Mapping of variables from our data

	**FRAIL-NH variables**^**8**^			**Corresponding variable from our data**		
**Score**	**0**	**1**	**2**	**0**	**1**	**2**
*FRAIL-NH component*						
Fatigue	No	Yes	PHQ-9		GDS15	
			≥10	0–5	6–9	≥10
Resistance	Independent transfer	Set up	Physical help	FIM (Bed, chair, and wheelchair transfer)		
				7	5–6	1–4
Ambulation	Independent	Assistive device	Not able	FIM (Walking and wheelchair)		
				7	5–6	1–4
Incontinence	None	Bladder	Bowel	FIM (Bladder or Bowel)	FIM (Bladder)	FIM (Bowel)
				FIM (Bladder) ≥6 and FIM (Bowel) ≥6	1–5	1–5
Weight loss	None	≥5% in 3 months	≥10% in 6 months	MNA-SF (B) No weight loss	MNA-SF (B) Other	-
Nutritional approach	Regular diet	Mechanically altered	Feeding tube	FOIS		
				Level 7	Levels 4–6	Levels 1–3
Help with dressing	Independent	Set up	Physical help	FIM (Dressing upper or lower body)		
				6–7	5	1–4

PHQ-9, Patient Health Questionnaire-9; GDS15, Geriatric Depression Scale 15; FIM, Functional Independence Measure; MNA-SF, Mini Nutrition Assessment — Short Form; FOIS, Functional Oral Intake Scale.

The GDS15 and MMSE were administered by a psychologist during patient interviews. The FIM was administered by a physical therapist on CICW admission. The MNA-SF and FOIS were completed by a nurse according to the information obtained from the electronic medical records. The information was collected on CICW admission.

### Data Collection

In addition to the above, we collected the following data: 1) nursing home admission following CICW discharge (yes vs. no); 2) transfer to a different hospital or death during CICW stay; 3) length of CICW stay; 4) living and economic status before CICW admission, based on interview with participants or their family members; 5) MMSE score, based on cognitive function assessment by psychologists using the Japanese version of the MMSE (24); and 6) primary illness for hospitalization.

### Statistical Analysis

Participants were divided into three groups based on the frailty status, and their characteristics were compared. In addition, we divided them into two subgroups based on whether or not they were admitted to a nursing home following CICW discharge and analyzed the risk of nursing home admission according to the frailty status.

Participants’ characteristics were presented as mean values, median values, or proportions according to the frailty status. The trend of measurements was tested by regression analysis and the Chi-square test. The person-days value for each measurement was calculated as the time to CICW discharge, transfer to a different hospital, or death. Nursing home admission event-free curves were derived using the Kaplan-Meier method and compared by the log-rank test.

The hazard ratios (HRs) and 95% confidence intervals (CIs) of nursing home admission were calculated according to the FRAIL-NH categories using the Cox proportional hazards models with reference to the robust group. In the multivariate adjusted model, we adjusted for age, sex, MNA-SF score (<8, 8–11, or ≥12), MMSE score (≤23 or >23), living status (0=not alone or 1=living alone), and economic status (0=not poverty or 1=poverty). Supplementary, we separated two groups at the median value of the length of CICW stay and calculated HRs and 95%CI of nursing home admission for each group.

Statistical analysis was performed using STATA (Stata/SE 17.0; Stata Corp., College Station, TX, U.S.A.). Two-tailed p<.05 was considered statistically significant.

## Results

In the comparison according to the frailty status, participants with frail status were older, had longer CICW length of stay, worse nutritional status, lower MMSE scores, more depressive symptoms, and more commonly admitted to a nursing home than those in the other two groups (Table 2).

**Table 2:** Baseline characteristics of the study population stratified by frailty status

	**Robust**	**Prefrail**	**Frail**	**p for trend**
	**N=258**	**N=97**	**N=195**	
Age, years, mean (SD)	81.9 (6.3)	83.9 (7.5)	83.6 (7.0)	.009
Women, n (%)	80 (31.0)	24 (24.7)	68 (34.9)	.90
Nursing home admission, n (%)	25 (9.7)	25 (25.8)	68 (34.9)	<.001
Length of CICW stay, days, median (IQR)	36.0 (27.0–49.0)	43.0 (30.0–54.0)	45.0 (35.0–55.0)	<.001
MNA-SF, n (%)				
8–11	161 (62.4)	45 (46.4)	50 (25.6)	.005
12–14	21 (8.1)	5 (5.2)	3 (1.5)	<.001
MMSE ≤23, n (%)	82 (31.8)	63 (64.9)	141 (72.3)	<.001
GDS15, mean (SD)	5.2 (3.4)	7.3 (3.5)	8.2 (3.8)	<.001
Living alone, n (%)	81 (31.4)	31 (32.0)	26 (13.3)	.001
Economic distress, n (%)	16 (6.2)	11 (11.3)	18 (9.2)	.112
Causal diseases, n (%)				
Malignancy	9(3.5)	2(2.1)	4(2.1)	.303
Cardiovascular disease	34(13.2)	13(13.4)	14(7.2)	.142
Infection	8(3.1)	4(4.1)	20(10.3)	.011
Orthostatic disease	157(60.9)	54(55.7)	107(54.9)	.176
Neuropsychological disorders	10(3.9)	8(8.3)	14(7.2)	.067
Other diseases	40(15.5)	16(16.5)	36(18.5)	.470

SD, standard deviation; CICW, Community-based integrated care ward; IQR, interquartile range; MNA-SF, Mini Nutrition Assessment — Short Form; MMSE, Mini-Mental State Examination; GDS15, Geriatric Depression Scale 15.

**Figure 2:**
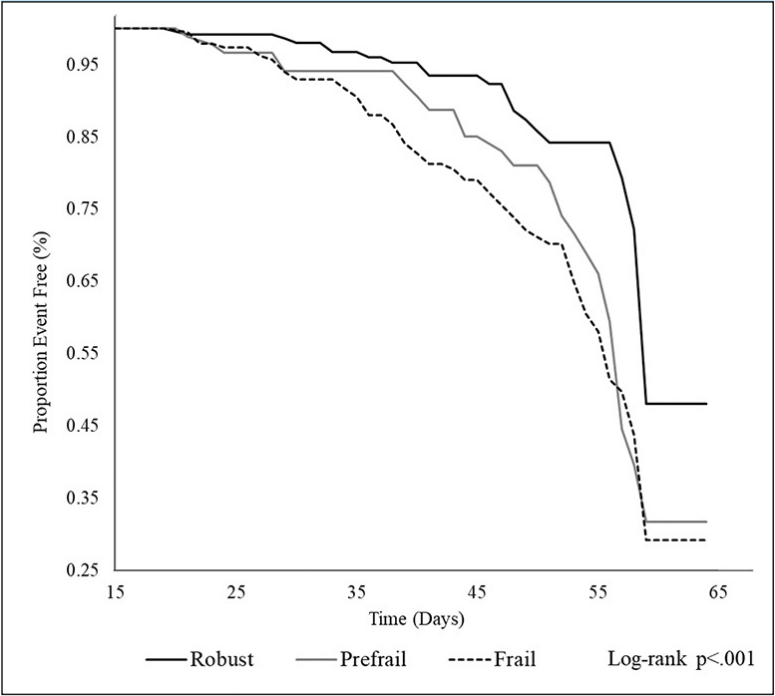
Kaplan-Meier curves for each frail category for the incidence of nursing home admission

In the multivariate analysis (Table 3), the frail group (HR, 2.22; 95% CI 1.32–3.76) and the prefrail group (HR, 1.69; 95% CI 0.95–3.00) had a higher risk of nursing home admission than robust group. The Kaplan-Meier curves also showed that the frail group had an increased risk of nursing home admission (Figure 2). In supplemental analysis, among short staying group, the HRs (95% CI) were 1.41 (0.49–1.09) for prefrail and 3.82 (1.56–9.34) for frail in model 3. In the long staying group, hazard ratios (95%CI) were 1.76 (0.88–3.51) for prefrail and 1.73 (0.90–3.34) for frail (Supplemental Table 1.

**Table 3:** Hazard ratios (95% confidence intervals) of nursing home admission compared to non-nursing home admission according to frailty status

	**Robust**	**Prefrail**	**Frail**
No. at risk	258	97	195
No. of cases	25	25	68
Pearson-days	9680	4022	8554
Non-adjusted HR	Reference	2.08 (1.20–3.63)	2.51 (1.58–3.97)
Age-sex adjusted HR	Reference	1.80 (1.02–3.15)	2.26 (1.42–3.59)
Multivariable-adjusted HR*	Reference	1.69 (0.95–3.00)	2.22 (1.32–3.76)

HR, hazard ratio. *Multivariable-adjusted HRs were adjusted for age, sex, Mini Nutrition Assessment — Short Form scores, Mini-Mental State Examination scores, living alone, and economic distress.

## Discussion

In this study, we showed that the FRAIL-NH score could predict nursing home admission among older adults admitted to post-acute care CICWs although we retrospectively calculated the FRAIL-NH scores.

More than 50% of our study participants had declined cognitive function and had orthostatic disease, affecting their mobility. In an observational study that included 500 older adults in a subacute geriatric ward, 35% of the participants were not assessed by Fried’s criteria (25), as they had cognitive impairment or difficulty with ADLs. The FRAIL-NH scale was developed to assess the frailty status in nursing home residents who have some difficulty in ADLs (8). Therefore, we believe that using the FRAIL-NH scale for patients in CICW was appropriate.

Frailty assessed by the FRAIL-NH scale has been associated with adverse health outcomes (11), comorbidities (26), and mortality (27–29) in nursing home residents, and with institutionalization (30), and mortality (30, 31) in patients. A prospective cohort study of 210 inpatients aged 65 years and older in a tertiary hospital for acute care found that frailty, assessed by the FRAIL-NH scale, was related to institutionalization or death after 6 months or 1 year (30). The odds ratio (95% CI) of institutionalization or death with reference to the non-frail inpatients was 4.98 (1.45–17.13) after 6 month and 6.03 (2.01–18.09) after 1 year. Although the participants in our study were inpatients in a post-acute care institution, our results were similar to those reported in the above study, suggesting that the FRAIL-NH scale could be useful in post-acute inpatient older adults.

In the present study, we calculated the FRAIL-NH scores retrospectively and the criteria we used slightly differed from those in the original version. We used FIM for assessing resistance, ambulation and incontinence, and FOIS for assessing nutritional approach. As FIM and FOIS were evaluated in more detail than the FRAIL-NH original version, we believe that misclassification for using FIM and FOIS might be less when they are retrospectively scored. However, GDS15 for assessing fatigue and MNA-SF for assessing weight loss has been controversial. A positive correlation was observed between GDS15 and PHQ-9 scores (r=0.676; p<.001) (20). In addition, we used the following cutoff points for GDS15 scores: ≤5 for “No,” 6–9 for “Yes,” and ≥10 for “PHQ-9 ≥10,” according to a previous study (20). Regarding the MNA-SF, this tool comprises four categories of weight loss: no weight loss, weight loss between 1 and 3 kg, weight loss greater than 3 kg, and does not know. We assigned a score of 1 to these 3 categories and a score of 0 to the “no weight loss” category according to a previous studies (21, 22). We believe that these differences have little effect on the results.

The strength of our study is the high reliability of the results; medical professionals collected all theinformation and performed the assessments and examinations. However, it has some several limitations. First, because we excluded participants with low MMSE scores (≤9) or missing MMSE, and this was a single-center study, the generalizability of the results is limited. Second, the retrospective FRAIL-NH scoring may have promoted scoring inaccuracies for some components of the FRAIL-NH scale. However, as the assessment tools we used, except the GDS-15 and MNA-SF, were fitted to the FRAIL-NH scale, it is unlikely that misclassification has occurred. The average age of the participants in this study was over 80 years; hence when they suffer from acute illnesses, they often lead to temporary or permanent disability. When assessing frailty during this period, the phenotype model (including FRAIL) may not lead to useful information due to ceiling effects. In this regard, the FRAIL-NH includes basic ADL items and their ratings; hence, more information can be collected when applied at the post-acute phase (an intermediate stage between the hospital and patients’ house). In this study, it was not possible to investigate whether the use of FRAIL-NH had an effect on the content of treatment, including rehabilitation, thus warranting further research.

## Conclusions and Implications

The FRAIL-NH scores are beneficial in predicting nursing home admission among older patients in post-acute care settings. The FRAIL-NH is effective in determing frailty status of older adults with difficulty in performing ADLs and is predictive of adverse health outcomes. Hence, patient assessment based on the FRAIL-NH scale may help clinical staff to effectively plan care and accordingly consider preparation and support for life after discharge. In the future, it is expected that the evaluation of FRAIL-NH will help to devise interventions that can lead to more appropriate care as well as clarify the effectiveness of such interventions.

## Electronic supplementary material

Supplemental Table 1. Hazard ratios (95% confidence intervals) of nursing home admission compared to non-nursing home admission according to frailty status stratified short and long staying CICW group.

## Notes

*Ethical Standards:* Ethical approval was obtained from the relevant Ethics Committee of Human Research of the National Center for Geriatrics and Gerontology, Japan (No. 830).
