# Supplementary material for: Prediction of Nursing Home Admission Using the FRAIL-NH Scale Among Older Adults in Post-Acute Care Settings
Source: J Nutr Health Aging. 2023 Mar 10;27(3):213–8. doi: 10.1007/s12603-023-1893-1 (PMC9999068; doi:10.1007/s12603-023-1893-1)
Supplement: Supplementary file 1 — Supplemental Table 1. Hazard ratios (95% confidence intervals) of nursing home admission compared to non-nursing home admission according to frailty status stratified short and long staying CICW group. [file 12603_2023_1893_MOESM1_ESM.docx]

Supplemental Table 1. Hazard ratios (95% confidence intervals) of nursing home admission compared to non-nursing home admission according to frailty status stratified short and long staying CICW group.

|  | Robust | Prefrail | | Frail | |
| --- | --- | --- | --- | --- | --- |
| Short staying group |  |  |  |  |  |
| No. at risk | 151 |  | 47 |  | 77 |
| No. of cases | 8 |  | 7 |  | 29 |
| Person-days | 4218 |  | 1377 |  | 2425 |
| Non-adjusted HR | Reference | 1.18 | (0.82–6.28) | 4.35 | (1.98–9.55) |
| Age-sex adjusted HR | Reference | 2.03 | (0.73–5.66) | 3.76 | (1.69–8.37) |
| Multivariable-adjusted HR^*^ | Reference | 1.41 | (0.49–4.09) | 3.82 | (1.56–9.34) |
| Long staying group |  |  |  |  |  |
| No. at risk | 107 |  | 50 |  | 118 |
| No. of cases | 17 |  | 18 |  | 39 |
| Person-days | 5462 |  | 2645 |  | 6129 |
| Non-adjusted HR | Reference | 2.08 | (1.07–4.03) | 1.99 | (1.13–3.53) |
| Age-sex adjusted HR | Reference | 1.86 | (0.95–3.65) | 1.81 | (1.02–3.22) |
| Multivariable-adjusted HR* | Reference | 1.76 | (0.88–3.51) | 1.73 | (0.90–3.34) |

HR, hazard ratio.

*Multivariable-adjusted HRs were adjusted for age, sex, Mini Nutrition Assessment - Short Form scores, Mini-Mental State Examination scores, living alone, and economic distress.
